# Cost-effective method for computational prediction of thermal conductivity in optical materials based on cubic oxides

**DOI:** 10.1038/s41598-024-63302-6

**Published:** 2024-06-10

**Authors:** A. Santonocito, B. Patrizi, A. Pirri, M. Vannini, G. Toci

**Affiliations:** 1 Dipartimento di Chimica, Università di Pisa, Via Giuseppe Moruzzi 13, 56124 Pisa, Italy; 2 Istituto Nazionale di Ottica, Consiglio Nazionale delle Ricerche, INO-CNR, Via Madonna del Piano 10, 50019 Sesto Fiorentino, FI Italy; 3 European Laboratory for Non Linear Spectroscopy, LENS, Via Nello Carrara 1, 50019 Sesto Fiorentino, FI Italy; 4 Istituto di Fisica Applicata “N. Carrara”, Consiglio Nazionale delle Ricerche, IFAC-CNR, Via Madonna del Piano 10, 50019 Sesto Fiorentino, FI Italy

**Keywords:** Density functional theory, Slack equation, Klemens equation, Thermal conductivity, Thulium doped materials, Sesquioxide laser ceramics, Theoretical chemistry, Optics and photonics, Materials science, Materials for optics, Lasers, LEDs and light sources, Computational science

## Abstract

In this paper we report on a computationally cost-effective method designed to estimate the thermal conductivity of optical materials based on cubic oxide including mixed ones, i.e. solid solutions of different oxides. The proposed methodology take advantage from Density Functional Theory (DFT) calculations to extract essential structural parameters and elastic constants which represent the inputs for revised versions of Slack and Klemens equations relating thermal conductivity to elastic constants. Slack equation is modified by the introduction of a corrective factor that incorporates the Grüneisen parameter γ, while in the revised Klemens equation a distortion parameter $d$ accounting for the impact of point defects on lattice symmetry is added, which is a critical factor in determining thermal conductivity in optical materials with mixed compositions. The theoretical results were found in good agreement with experimental data, showing the reliability of our proposed methodology.

## Introduction

The thermal conductivity of a solid-state system quantifies the heat flux through the propagation of vibrational energy from one atom to adjacent atoms in the lattice without the transport of matter. In essence, it measures a material ability to conduct heat. Thermal transport properties are important for different categories of materials, including thermo-electrics, opto-electronics, photovoltaic and photo-electrochemical cells, batteries. The accurate knowledge of thermal properties is particularly crucial for the design of high-intensity laser systems, where an ideal gain material should exhibit high thermal conductivity to promote efficient heat removal and prevent thermal stresses^1^. The incorporation of lasing dopants ions, such as Yb^3+^^1–7^ or Tm^3+^^8,9^, into a host material (crystal or ceramics) leads to the formation of localized lattice defects, which can reduce the overall thermal conductivity of the material, eventually affecting its laser emission performance. The selection of a host material with a high intrinsic thermal conductivity is a critical consideration in the development of mixed laser ceramics, as it can mitigate the thermal degradation caused by the presence of these dopants. The material should possess sufficient thermal stability and conductivity even in the presence of high concentrations of dopant, so that it can maintain a high level of thermal conductivity during the demanding conditions of laser operation.

Of course, thermal properties of materials can be experimentally assessed by direct measurements, but this requires the availability of good quality and large samples and the execution of complex and time-consuming measurements. For this reason, the availability of accurate computational methods for the prediction of thermal properties is attracting attention as a convenient alternative to experimental properties screening, in particular when dealing with new, scarcely available and poorly characterized materials and compounds.

Currently, the prediction of thermal conductivity is based on three categories of methods: Anharmonic Lattice Dynamics (ALD) in combination with phonon transport calculation using the Boltzmann transport equation (BTE) and Fourier’s law^10^; Equilibrium Molecular Dynamics (EMD) using the Green– Kubo formula^11^ and the direct evaluation of the heat flux by Non-Equilibrium Molecular Dynamics (NEMD)^12,13^. These current state-of-the-art methods are computationally expensive and time-consuming, limiting their practical applicability especially for complex systems. This is due to the need to solve complex equations and perform multiple simulations. Therefore, there is a pressing need for more efficient methods that can provide accurate predictions of thermal conductivity for complex systems in a cost-effective manner.

In this study, we propose a novel computational approach for estimating the thermal conductivity of optical materials based on cubic oxides, involving a series of steps including Density Functional Theory (DFT) calculations to obtain structural parameters and elastic constants. These calculations provide the key inputs for refined versions of the Slack and Klemens equations, which are then employed to enhance the accuracy of lattice thermal conductivity predictions. Slack equation is modified by introducing a corrective factor that incorporates the Grüneisen parameter γ. The Grüneisen parameter takes into account the bonds anharmonicity. On the other hand, Klemens equation is revised by introducing a geometrical factor that accounts for the effects of point defects on lattice symmetry, which is a key factor in determining thermal conductivity of materials. In order to validate our method we have chosen two families of optically active materials, namely Y and Sc mixed sesquioxides, (Y,Sc)_2_O_3_, doped with Tm, and Lu-doped Yttrium Aluminum Garnet (YAG). This interest stems from a broader research activity devoted to assessing the optical, spectroscopic and laser properties of candidate laser materials.

More in detail, we tested the following classes of compositions:

- 5 at.% Tm^3+-^doped (Y,Sc)_2_O_3_ transparent ceramics with varying Sc^3+^ concentrations (0; 12.1, 25.2 and 49.8 at.%^14^.
- Lu-doped YAG crystals with varying Lu^3+^ concentrations (0, 33.3, 50.0, 66.7, 100 at.%)^15^.

For sake of simplicity, the Tm^3+^ doped mixed Y_2_O_3_/Sc_2_O_3_ ceramics at 12.1 at.% 25.1 at.% and 49.8 at.% of Sc_2_O_3_ will be named as Sc_12_, Sc_25_ and Sc_50_, while the Lu^3+^-doped mixed Y_3_Al_5_O_12_/Lu_3_Al_5_O_12_ crystals at 33.3 at.%, 50 at.%, 66,7 at.% of Lu_3_Al_5_O_12_ will be renamed as Lu_33_, Lu_50_, Lu_67_.

The structural parameters, elastic constants, and thermal conductivity values obtained for the analysed compounds by the proposed model have been compared to experimental data, showing good agreement across all the cases. This confirms the accuracy and robustness of the proposed methodology for predicting thermal conductivity in mixed composition materials. This work addresses a significant gap in the current literature, as there is currently a lack of reliable and cost-effective methods for routine thermal conductivity prediction in cubic oxides, used as laser and luminescent materials.

## Theoretical approach

### Ab initio calculations of lattice structures

The structural calculations were performed using the plane wave periodic DFT implemented in CASTEP^16^ employing the PBEsol exchange–correlation functional^17^ with Grimme D3 dispersion correction^18^. The ultrasoft pseudopotentials from the internal QC5 library of CASTEP were used for the Y, Tm, Lu, Al, Sc, and O atoms, with a plane wave cut-off of 410 eV, a Self-Consistent Field convergence threshold equal to ${10}^{-8} \text{eV}/\text{atom}$, a k-point grid with a fine k-point separation of 0.04 Å^−1^ and a convergence criterion for the maximum force component equal to 0.01 eV/Å. The Limited memory Broyden–Fletcher–Goldfarb–Shanno algorithm (LBFGS)^19^, which scales linearly with respect to the system size, was employed to perform the geometric optimization. This optimizer utilizes a limited number of inverse Hessian updates to construct a new Hessian, a process which requires significantly less computational effort than the traditional BFGS algorithm, which scales quadratically with the system size^20^.

Furthermore, Periodic Boundary Conditions (PBCs) were employed to account for the long-range periodicity inherent to crystalline solid materials.

We started our work from conventional unit cells belonging to the cubic space group $Ia\overline{3 }$ and containing 16 cell formula units (Z = 16) for the study of all the systems of our interest. The optimization of the cell is performed by searching for the Potential Energy Surface (PES) local minimum (closest to the starting structure) by varying all the parameters of the cell, *i.e.* unconstrained model, obtaining the optimized sides and angles of the cell, as well as the coordinates of all the atoms. The aforementioned computational methodology enabled the calculation of the cell dimensions, bond lengths, and inter-ionic distances, as well as the determination of local structural distortions of the studied systems. Furthermore, the Vegard’s law was employed to estimate unit cell sides^21^ according to the following equation:

1 $${a}_{\text{C }}= x{a}_{\text{B}}+(1-x){a}_{\text{A}}$$

This empirical rule provides a description of the variation of lattice sides (${a}_{\text{i}}$) in a solid solution (i = C), which is composed of two components (A and B) with relative weights $x$ and $(1-x)$, respectively. Although this kind of empirical rules may provide useful insights it is important to note that they are not rigorously predictive and should be utilized with caution. However, in the absence of experimental data, they may serve as a useful point of comparison with results from ab initio calculations.

### Ab initio calculations of elastic constants

For the elastic constant calculations we used CASTEP software packages^16^ performing calculations at DFT level with PBEsol functional. The linear response of the stress vector to a given strain vector is described as:

2 $${\varvec{\upsigma}}=\text{C}\cdot {\varvec{\upvarepsilon}}$$

where ${\varvec{\upsigma}}$ and ${\varvec{\upvarepsilon}}$ are the symmetric stress and strain tensors respectively, and $\text{C}$ is the symmetric 6 × 6 matrix of elastic constants. In a cubic crystal, only three elements, C_11_, C_12_, and C_44_, are independent. To obtain the matrix $\text{C}$, small deformations, δ (of the order of 1% for cell sides and 2% for cell angles), are applied to the simulation cell along the strain vectors, and the resulting stress vectors are calculated. The maximum force and stress are set to 0.001 eV/Å and 0.001 eV/Å^3^ respectively, by employing the LBFGS optimizer. From the C_11_, C_12_, and C_44_ elastic constants, several important material properties can be calculated using the Voigt–Reuss–Hill scheme^22,23^. These properties include the Bulk modulus (*B*), the Voigt Shear modulus (G_V_), the Reuss Shear modulus (*G*_*R*_), the Voigt–Reuss–Hill Shear modulus (*G*), Young modulus (*E*), and Poisson ratio (υ), which can be computed using the following Eqs. (3–8):

3 $$B=\frac{{C}_{11}+2{C}_{12}}{3}$$

4 $${G}_{V}=\frac{{C}_{11}-{C}_{12}+3{C}_{44}}{5}$$

5 $${G}_{R}=\frac{{5(C}_{11}-{C}_{12})\cdot {C}_{44}}{{4C}_{44}+3{(C}_{11}-{C}_{12})}$$

6 $$G=\frac{{\text{G}}_{V}+{\text{G}}_{R}}{2}$$

7 $$E=\frac{9B\cdot G}{3B+G}$$

8 $$\upupsilon =\frac{\text{E}}{2G}-1$$

Using the *B* and *G* moduli, the following velocities can be calculated: longitudinal wave velocity (${v}_{L}$),^24,25^ shear wave velocity (${v}_{S}$),^24,25^ and sound velocity (${v}_{a}$).^22,24–26^ Furthermore, the Grüneisen parameter (γ)^27^, the Debye temperature (${\uptheta }_{\text{D}})$^24,25^ and acoustic Debye temperature (${\uptheta }_{\text{a}}$)^28^ can be determined from well-established Eqs. (9–14):

9 $${v}_{L}={\left(\frac{3B+4G}{3\rho }\right)}^\frac{1}{2}={\left(\frac{E\left(1-\upupsilon \right)}{\rho \left(1+\upupsilon \right)\left(1-2\upupsilon \right)}\right)}^\frac{1}{2}$$

10 $${v}_{S}={\left(\frac{G}{\rho }\right)}^\frac{1}{2}$$

11 $${v}_{a}={\left(\frac{1}{3}\left(\frac{2}{{{v}_{S}}^{3}}+\frac{1}{{{v}_{L}}^{3}}\right)\right)}^{-\frac{1}{3}}$$

12 $$\upgamma =-\frac{\partial \text{ln}(\omega )}{\partial \text{ln}(V)}=\frac{\beta B{V}_{m}}{{C}_{v}}$$

13 $${\uptheta }_{\text{D}}=\frac{h}{2k}{v}_{a}{\left(\frac{6}{\pi }\frac{n}{V}\right)}^\frac{1}{3}$$

14 $${\uptheta }_{\text{a}}=\frac{{\uptheta }_{\text{D}}}{\sqrt[3]{{n}_{p}}}$$

where $\uprho$ is the density of the system, $n$ is the number of atoms in the unit cell, ${n}_{p}$ is the number of atoms in the primitive cell, β is the volume thermal expansion coefficient, V_m_ is the molar volume, C_v_ is the molar heat capacity, $h$ is the Plank constant and $k$ is the Boltzmann constant. All these quantities are expressed in International System units (SI)*.*

Regarding the Grüneisen parameter $\upgamma$ (which takes into account of the average anharmonicity of the bonds), we preferred not to use the original Grüneisen formula (12), because the evaluation of Cv requires the computational evaluation of the whole phonon spectrum, which is a computational intensive and demanding task. Rather we took advantage from the Leontiev’s approach, further elaborated by Belomestnykh, and Sanditov in References^29–31^ that established a relationship between the average value of the Grüneisen parameter γ that characterizes the degree of anharmonicity of interatomic forces and the velocities of sound in an isotropic, spatially unbounded elastic medium, i.e.:

15 $$\upgamma =\frac{9}{2}\left(\frac{{{v}_{L}}^{2}-\frac{4}{3}{{v}_{S}}^{2}}{{{v}_{L}}^{2}+2{{v}_{S}}^{2}}\right)$$

Using this formula, γ can be derived from experimental data of sound velocity, which are much more readily available than phonon spectra. The relationship above was verified over a broad range of crystalline solid^29^, and its validity was recently reviewed in^32^. Besides, it was validated against a broad set of experimental data^29,31,32^ so we deemed it can be reliably adopted as a proxy for the original Grüneisen formula (Eq. 12).

### Models for thermal conductivity prediction

#### Pure crystalline solids

In a crystalline solid, thermal energy can be conveyed through the motion of both electrons and phonons, i.e. quasiparticles which describe lattice vibrations. In insulators and semiconductors, lacking free charge carriers, thermal conduction is solely mediated by phonons. As it is well known, while a hypothetical, idealized crystal would possess an infinite lattice thermal conductivity, real solids are inevitably imperfect and subject to anharmonic oscillations. These factors have a direct impact on the lifespan and behavior of phonons, ultimately affecting the thermal conductivity of the material.

**Figure 1:**
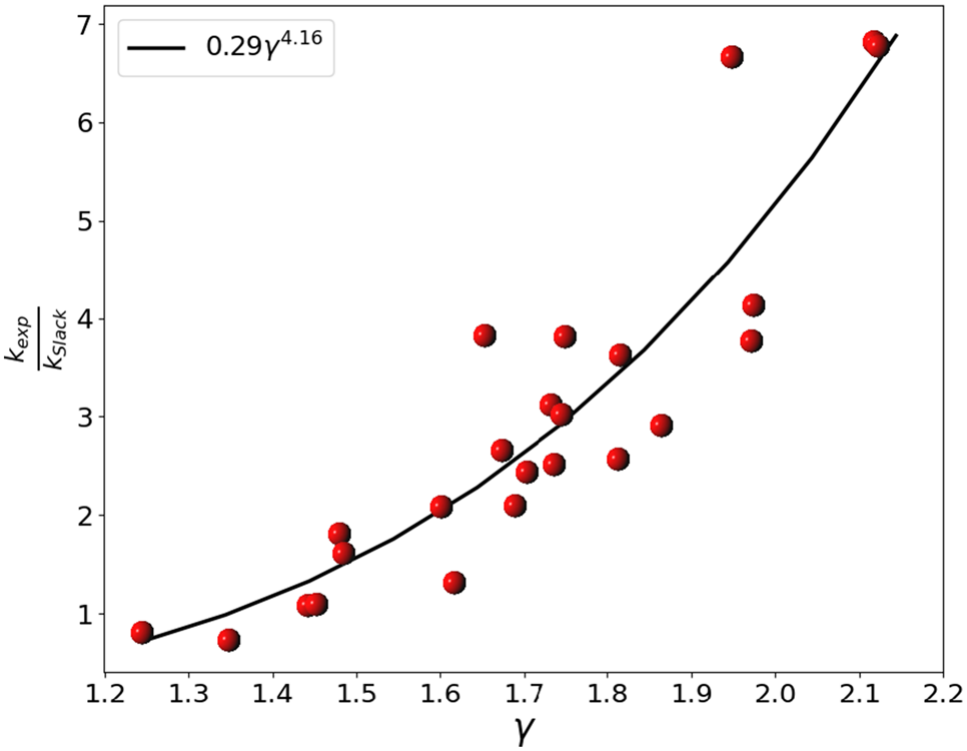
Fitting (black line) of the ratio between the experimental and the Slack calculated thermal conductivities (red points) as a function of the Grüneisen parameters. The exact expression for the fitting curve is 0.289003 $\cdot {\gamma }^{4.156157}.$

In literature we can find a multitude of approaches for calculating lattice thermal conductivity. One of the most popular analytical model of lattice thermal conductivity is the Slack equation^33^:

16 $${k}_{Slack}(T)=\frac{A}{1-0.514{\gamma }^{-1}+0.228{\gamma }^{-2}}\cdot {\left(\frac{2\pi k{\uptheta }_{a}}{h}\right)}^{2}\cdot \frac{2\pi k{M}_{av}{{V}_{p}}^\frac{1}{3}}{h{\gamma }^{2}}\cdot \frac{{\uptheta }_{a}}{T}$$

where *T* is the temperature, $A=\left(0.849\cdot 3\sqrt[3]{4}\right)/(20{\pi }^{3})$ is an empirical parameter, ${M}_{av}$ is the mass in *kg* of the unit formula divided by the number of constituent atoms and ${{V}_{p}}^\frac{1}{3}={\delta \cdot {n}_{p}}^\frac{1}{3}$ with ${\delta }^{3}$ the volume per atom in *m*^3^. The Slack equation is a theoretical model that describes the thermal conductivity of a material at various temperatures. It assumes that three-phonon scattering is the dominant mechanism of heat transfer, meaning that the heat is transferred through the vibrations of the material atoms. This equation considers the average mass of the atoms in the material, the speed of sound in the material, and the Debye temperature, which is a measure of the thermal excitation of the material lattice vibrations. Although the Slack formula depends on the empirical parameter A (see Eq. 16), it was chosen as a reference for the calculation of the thermal conductivities of pure compounds because it is one of the most widely used approximations for the estimation of the thermal conductivity of non-metallic compounds. However, in this work we propose a functional form for *A* depending on γ, derived from the analysis of the thermal properties of a set of compounds based on cubic oxides reported in Table 1. In particular, the experimental values of the thermal conductivity (k_exp_) of these compounds have been obtained from literature while $\upgamma$ values for each compounds have been calculated from the parameters *B* and *G* reported in Material Project Database^34^. As it is possible to see by inspecting Fig. 1 there is a correlation between k_exp_/k_Slack_ and γ for the cubic oxides dataset analysed. This correlation can be modeled as a power function of γ giving rise to a revised Slack equation.

**Table 1:** Data used for the calculation of the corrective term of Slack equation in cubic oxides. The values of $\gamma$ have been calculated by the bulk (*B*) and shear modulus (*G*)^34^. The thermal conductivities are expressed in ${\text{Wm}}^{-1}{\text{K}}^{-1}$.

Compounds	k_exp_	k_Slack_	γ	k_exp_/k_Slack_
Y_2_O_3_	12.72^35^	3.520	1.815	3.614
Sc_2_O_3_	17.00^36^	5.478	1.733	3.103
Lu_2_O_3_	12.50^37^	3.285	1.653	3.806
In_2_O_3_	13.10^38^	1.970	1.948	6.649
Gd_2_O_3_	6.20^39^	2.137	1.864	2.901
Er_2_O_3_	6.50^39^	2.541	1.812	2.558
Tm_2_O_3_	9.64^40^	2.542	1.749	3.793
Y_3_Al_5_O_12_	12.90^15^	7.169	1.479	1.799
Lu_3_Al_5_O_12_	9.60^15^	6.006	1.484	1.598
Y_3_Fe_5_O_12_	7.40^41^	2.959	1.736	2.501
Ca_3_Al_2_Si_3_O_12_	7.20^42^	6.736	1.442	1.069
Yb_3_Al_5_O_12_	6.90^42^	6.416	1.452	1.076
Y_3_Ga_5_O_12_	9.00^42^	3.406	1.673	2.642
Gd_3_Ga_5_O_12_	9.00^42^	2.989	1.745	3.011
Mg_3_Al_2_Si_3_O_12_	5.55^42^	4.263	1.616	1.302
Gd_3_Al_5_O_12_	9.80^42^	4.037	1.703	2.427
Er_3_Al_5_O_12_	7.60^42^	3.670	1.601	2.071
Ho_3_Al_5_O_12_	9.30^42^	4.459	1.689	2.086
Ho_3_Ga_5_O_12_	6.50^42^	1.733	1.972	3.751
Er_3_Ga_5_O_12_	7.00^42^	1.701	1.974	4.116
Yb_3_Ga_5_O_12_	6.50^42^	0.956	2.118	6.801
CaO	27.00^43^	37.481	1.347	0.720
MgO	60.00^43^	75.057	1.244	0.799
NiO	50.00^44^	7.401	2.122	6.756

The revised version of Slack equation has been applied to the dataset of the compounds reported in Table 1 which reports the experimental (k_exp_) and the Slack (k_Slack_) thermal conductivities employed for the estimation of the corrective term *A*, as a function of γ calculated as reported in Eq. 16.

Thus, the modified Slack equation for cubic oxides becomes:

17 $${k}_{rev.Slack}(T)=\frac{0.289003{\cdot \gamma }^{4.156157}\cdot \left(0.849\cdot 3\sqrt[3]{4}\right)}{20{\pi }^{3}\cdot \left(1-0.514{\gamma }^{-1}+0.228{\gamma }^{-2}\right)}\cdot {\left(\frac{2\pi k{\uptheta }_{a}}{h}\right)}^{2}\cdot \frac{2\pi k{M}_{av}{{V}_{p}}^\frac{1}{3}}{h{\gamma }^{2}}\cdot \frac{{\uptheta }_{a}}{T}$$

The $0.289003{\cdot \gamma }^{4.156157}$ correction considers the influence of phonon scattering processes due to anharmonicity, characteristic of the specific material symmetry, which are not fully captured by the basic Slack model. This opens the possibility of using the Grüneisen’s parameter (which depend on crystal symmetry and chemical composition) to obtain specific multiplicative function for different symmetry classes of materials.

This approach is reasonable because this parameter describes how the *i*-th phonon frequencies (ω_i_) are affected by the cell volume (V), indeed:

18 $$\gamma =-\frac{V}{{\omega }_{i}}\frac{\partial {\omega }_{i}}{\partial V}$$

Equation 18 shows that γ is influenced by the underlying crystal symmetry (i.e. the vibrational modes symmetry) and by the chemical composition. Eq. 17 has been applied to the compounds reported in Table 2 demonstrating a substantial improvement in predictive accuracy with respect to the classic Slack equation. The revised Slack equation exhibits a markedly higher Pearson coefficient (R^2^ = 0.944), a mean relative percentage error of 19.539%, a higher correlation coefficient (μ = 0.972) and a considerably lower Mean Squared Error (MSE = 9.987) with respect to the original Slack equation (R^2^ = 0.620, μ = 0.788, MSE = 122.862, and mean relative percentage error of 56.348%). These results highlight the increased precision and accuracy achieved through the refined Slack equation.

**Table 2:** Experimental (k_exp_), original version (k_Slack_), revised Slack (k_rev.Slack_) thermal conductivities expressed in ${\text{Wm}}^{-1}{\text{K}}^{-1}$ and the relative percentage error Δ%.

Compounds	k_exp_	k_Slack_	Δ%	k_rev.Slack_	Δ%
Y_2_O_3_	12.72^35^	3.520	72.330	12.123	4.690
Sc_2_O_3_	17.00^36^	5.478	67.775	15.557	8.489
Lu_2_O_3_	12.50^37^	3.285	73.724	7.662	38.706
In_2_O_3_	13.10^38^	1.970	84.960	9.095	30.572
Gd_2_O_3_	6.20^39^	2.137	65.532	8.217	32.535
Er_2_O_3_	6.50^39^	2.541	60.905	8.679	33.529
Tm_2_O_3_	9.64^40^	2.542	73.634	7.506	22.137
Y_3_Al_5_O_12_	12.90^15^	7.169	44.424	10.551	18.212
Lu_3_Al_5_O_12_	9.60^15^	6.006	37.434	8.961	6.651
Y_3_Fe_5_O_12_	7.40^41^	2.959	60.015	8.461	14.340
Ca_3_Al_2_Si_3_O_12_	7.20^42^	6.736	6.439	8.905	23.682
Yb_3_Al_5_O_12_	6.90^42^	6.416	7.022	8.733	26.566
Y_3_Ga_5_O_12_	9.00^42^	3.406	62.154	8.349	7.238
Gd_3_Ga_5_O_12_	9.00^42^	2.989	66.786	8.744	2.842
Mg_3_Al_2_Si_3_O_12_	5.55^42^	4.263	23.191	9.049	63.040
Gd_3_Al_5_O_12_	9.80^42^	4.037	58.803	10.668	8.852
Er_3_Al_5_O_12_	7.60^42^	3.670	51.705	7.497	1.359
Ho_3_Al_5_O_12_	9.30^42^	4.459	52.051	11.374	22.301
Ho_3_Ga_5_O_12_	6.50^42^	1.733	73.342	8.419	29.516
Er_3_Ga_5_O_12_	7.00^42^	1.701	75.706	8.302	18.604
Yb_3_Ga_5_O_12_	6.50^42^	0.956	85.297	6.250	3.840
CaO	27.00^43^	37.481	38.820	37.370	38.407
MgO	60.00^43^	75.057	25.095	53.768	10.386
NiO	50.00^44^	7.401	85.199	48.772	2.455

#### Doped solids

The presence of dopant ions in optical materials, such as crystals and ceramics, leads to the creation of lattice defects, which can in turn affect the thermal conductivity of the material. For this reason, we developed a method to predict thermal conductivity in this kind of doped materials. The method is based on a revised version of Klemens formula.

**Figure 2:**
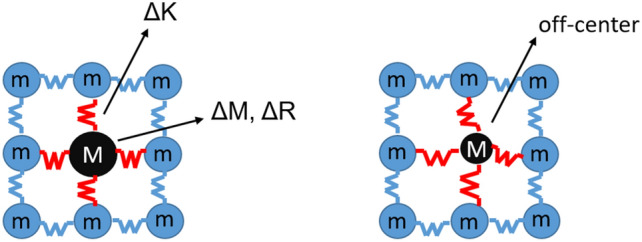
Left: Schematic representation of lattice perturbation due to the presence of point defects i.e. mass difference (ΔM $=|M-m|$), constant harmonic force difference (ΔK), radius difference (ΔR); right: Schematic representation of the symmetry loss of the site.

As it is well known, the original version of Klemens equation^45–47^ predicts the thermal conductivity of lattice having some point defects considering only the mass variation on phonon transport (see Eq. 19). The Klemens equation can be expressed as:

19 $${{k}_{L}}_{\text{K}}=\frac{{k}_{m}\cdot {\text{tan}}^{-1}\left(u\right)}{u}$$

20 $$u= \sqrt{\frac{{\left(6\cdot {\pi }^{5}\cdot {{V}_{0}}^{2}\right)}^\frac{1}{3}\cdot \Gamma \cdot {k}_{m}}{2\cdot k\cdot {v}_{a}}}$$

where ${V}_{0}$ is the volume per atom, $\Gamma$ is the scattering parameter (see Eqs. 21–23) and ${k}_{m}=\left(1-x\right)\cdot {k}_{0}+x\cdot {k}_{100}$ is a corrective term in which $x$ is the concentration of dopant atoms, ${k}_{0}$ and ${k}_{100}$ are thermal conductivities of solids with the compositions $x=0$ and $x=1$ respectively. At each composition, the parameters ${k}_{m}$, ${v}_{a}$, and ${V}_{0}$ undergo gradual adjustments through linear interpolation, using the properties of pure host *A* and *B*. Klemens firstly proposed the estimation of the scattering parameter Γ through the following Eq. ^48^:

21 $$\Gamma ={\sum }_{i=1}^{k}{c}_{i}{\left(\frac{{M}_{i}-M}{M}\right)}^{2}$$

where the scattering parameter $\Gamma$ is essentially the average mass variance in the system, ${\left({M}_{i}-M\right)}^{2}$, relative to the square of the average mass ${M}^{2}$. Another term which can consider the point defect system perturbation is the average change in atomic radius (ΔR) and the variation of the harmonic force constant (ΔK) mainly due to a change of the bond strengths with respect to the non-perturbed sites. Indeed, the introduction of dopant ions creates lattice point defects, which perturb the Hamiltonian of the system, inducing a rearrangement of the phonon probability density because of changes in the site mass (ΔM), harmonic force constants (ΔK), ionic radii (ΔR), and crystal symmetry, as depicted in Fig. 2 (left). These perturbations affect the potential energy of the material, enhancing phonon scattering due to a shortened mean free path, and ultimately decreasing the thermal conductivity. ΔM, ΔK, and ΔR are responsible for specific contributions: ΔM changes the material density, ΔK changes the lattice stiffness and ΔR changes the distance between atoms, all together influence the phonon propagation and thermal conductivity.

The interplay between these factors creates a complex picture of the material thermal behavior.

The relationships between force constants and atomic volumes could be described by a phenomenological fitting parameter. These were initially proposed by Abeles^49^ and then modified by Wan et al.^50^ based on the derivation of Klemens and Callaway:

22 $${\Gamma }_{i}={c}_{i}\left\{{\left(\frac{{M}_{i}-M}{M}\right)}^{2}+\frac{2}{9}\cdot {\left(6.4\cdot \gamma \cdot \frac{1+\upupsilon }{1-\upupsilon }\right)}^{2}\cdot {\left(\frac{{R}_{i}-R}{R}\right)}^{2}\right\}$$

where ${\Gamma }_{i}$ considers the defects due to the atoms of the chemical specie $i$.

For a mixed compound containing several defect atoms the total scattering parameter is given by:

23 $$\Gamma ={\sum }_{i=1}^{k}{\Gamma }_{i}$$

We insert a term in $\Gamma$, which considers the symmetry loss due to local distortions, see Fig. 2 (right), as the concentration of dopant cations increases and the cohesion of the material changes.

This corrective term is a power function of the parameter $d$:

24 $$d=\frac{\left|{\overrightarrow{\Delta R}}_{i}\right|}{{a}_{C}}\cdot \frac{2\sigma }{{{\varvec{a}}}_{\text{A}}+{{\varvec{a}}}_{\text{B}}}$$

where $\left|{\overrightarrow{\Delta R}}_{i}\right|$ with *i* = *A or B* is the average displacement of the atoms of the partially doped system *C*, in comparison to the undoped host lattice *A or B* respectively; $\sigma$ is the average bond length, for the compound under consideration. The variables ${{\varvec{a}}}_{{\varvec{i}}}$ are the average side of the cell with *i* $=A$, $B$, $C$ referring respectively to the pure compounds $A$, $B$ and to the mixed compositions samples, $C,$ with weight $x$ for $A$ and $(1-x)$ for $B$, that is *a*_*C*_ = *xa*_*A*_ + *(1-x)a*_*B*_. The term $\frac{{{\varvec{a}}}_{\text{A}}+{{\varvec{a}}}_{\text{B}}}{2}$ is used as a normalization factor ensuring the consistency of the model and comparability across different materials.

Accordingly, ${\Gamma }_{i}$ can be written as:

25 $${\Gamma }_{i}={c}_{i}\left\{{\left(\frac{{M}_{i}-M}{M}\right)}^{2}+\frac{2}{9}\cdot {\left(6.4\cdot \gamma \cdot \frac{1+\upupsilon }{1-\upupsilon }\right)}^{2}\cdot {\left(\frac{{R}_{i}-R}{R}\right)}^{2}+(a\cdot {d}^{r})\right\}$$

All the corrective terms in the case of pure compound assume a null value. Equation 25 shows that the term $d$ is integrated into ${\Gamma }_{i}$ as part of a power function with coefficient $a$ and exponent $r$. The parameters $a$ and $r$ are extrapolated from the fitting of the difference between the value of $\Gamma$ deriving from experimental values and ${\Gamma }_{i}$ obtained from original Klemens equation form. Taking advantage from the corrective parameter $d$, the proposed revised Klemens model considers the impact of point defects on lattice symmetry and the impact of the overall atoms displacement as depicted in Fig. 2.

The modified Klemens equation is thus given by:

26 $${{k}_{L}}_{\text{K}}=\frac{{k}_{m}\cdot {\text{tan}}^{-1}\left(\sqrt{\frac{{\left(6\cdot {\pi }^{5}\cdot {{V}_{0}}^{2}\right)}^\frac{1}{3}\sum_{i}{c}_{i}\left\{{\left(\frac{{M}_{i}-M}{M}\right)}^{2}+\frac{2}{9}\cdot {\left(6.4\cdot \gamma \cdot \frac{1+\upupsilon }{1-\upupsilon }\right)}^{2}\cdot {\left(\frac{{R}_{i}-R}{R}\right)}^{2}+(a\cdot {d}^{r})\right\}\cdot {k}_{m}}{2\cdot k\cdot {v}_{a}}}\right)}{\sqrt{\frac{{\left(6\cdot {\pi }^{5}\cdot {{V}_{0}}^{2}\right)}^\frac{1}{3}\cdot \sum_{i}{c}_{i}\left\{{\left(\frac{{M}_{i}-M}{M}\right)}^{2}+\frac{2}{9}\cdot {\left(6.4\cdot \gamma \cdot \frac{1+\upupsilon }{1-\upupsilon }\right)}^{2}\cdot {\left(\frac{{R}_{i}-R}{R}\right)}^{2}+(a\cdot {d}^{r})\right\}\cdot {k}_{m}}{2\cdot k\cdot {v}_{a}}}}$$

## Cost effective method for thermal conductivity prediction: overview and applications to mixed composition laser materials

This method provides a cost-effective approach for lattice thermal conductivity predictions in optical materials based on cubic oxides.

We start from the pre-relaxed pure compound structure from the Materials Project database^51^ that is refined through the structural relaxation in the DFT framework with PBEsol functional and Grimme D3 dispersion correction, providing a high-accurate representation of the system. The elastic constants, which characterize the material mechanical properties, are calculated using the same *ab initio* approach, providing a comprehensive and internally consistent set of parameters. These elastic constants are then utilized to calculate the Grüneisen parameter, Debye acoustic temperature, and acoustic velocity, which collectively characterize the thermal properties of the material (according to Eqs. 9–15). To achieve a more accurate prediction of the thermal conductivities of cubic oxide optical materials, a modified Slack equation is employed, which incorporates the multiplicative corrective factor of γ, as reported in Eq. 16. Regarding the doped compounds, starting from the pure compound optimized geometry, a doped structure is generated, and DFT-PBEsol relaxation is employed to refine its atomic coordinates. The refined structure is then utilized to calculate the corrective geometric factor, which quantifies the influence of defects on the crystal lattice symmetry and material cohesion. In order to accurately model the effects due to the point defects on thermal conductivity, a modified Klemens equation is applied (see Eq. 26). This equation is utilized to accurately predict the impact of point defects on thermal conductivity by also considering the distortion parameter *d*, which is a quantitative measure of the deviations from the ideal lattice symmetry due to the presence of defects.

**Figure 3:**
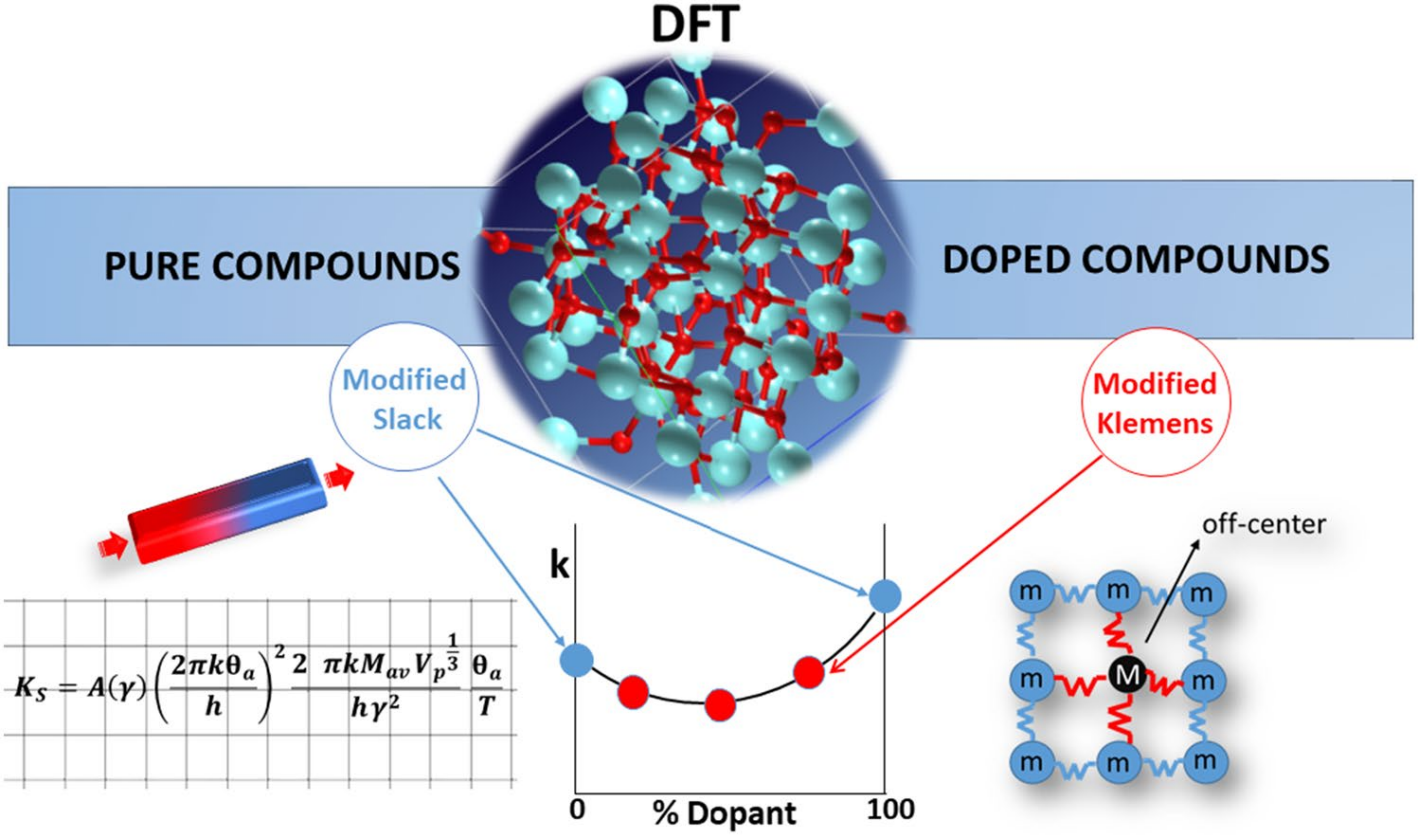
Schematic representation of the method proposed for the prediction of thermal conductivity in both pure and doped cubic oxides.

More precisely, the multiplicative factor *a* and the exponent *r* results from the best fit obtained by the minimization of the MSE between the experimental and calculated thermal conductivity values of both mixed compositions garnets and sesquioxides series of samples. Figure 3 reports in a schematic way the calculation steps of our method.

In the following paragraphs we test our method on two classes of cubic oxides materials with different compositions. In the first case study we estimate thermal conductivity in binary garnet systems while in the second case we analyze a more complex series of ternary mixture of sesquioxides both employed as laser materials.

### Prediction of thermal conductivity in Lu-doped yttrium aluminum garnet

#### Calculation of the lattice parameters

We calculated structural parameters for the series YAG, Lu_33_, Lu_50_, Lu_67_ and LuAG.

**Figure 4:**
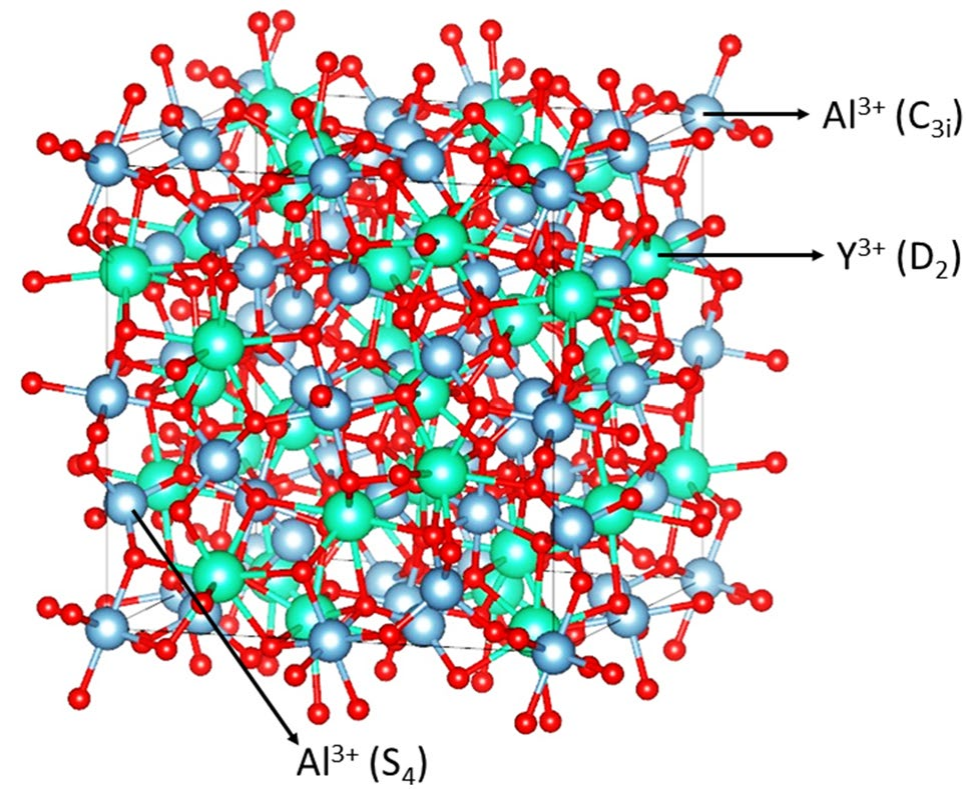
Lattice structure of YAG_._ Yttrium sites with symmetry D_2_ are colored in cyan while Aluminum sites with symmetries C_3i_ and S_4_ in light blue, the oxygen atoms in red.

The crystal structures of YAG and LuAG are both cubic, exhibiting the Ia3̅d space group symmetry (see Fig. 4). The conventional unit cell contains 8 formula units, *i.e.,* 160 atoms in total. There are 16 octahedrally coordinated Al^3+^ cations (site symmetry C_3i_), 24 tetrahedrally coordinated Al^3+^ cations (site symmetry S_4_), and 24 dodecahedrally coordinated Y^3+^ cations for YAG and 24 dodecahedrally coordinated Lu^3+^ cations for LuAG (site symmetry D_2_) and 96 O^2−^ anions occupying general non symmetric positions.

The arrangement of the ions leads to a specific type of chemical bonding and a distinctive distribution of phonon frequencies, which have a significant impact on the thermal transport properties of these materials. As already mentioned, the addition of Lu^3+^ dopants in YAG host introduces structural distortions and point defects (i.e. phonon scattering centers), leading to a decrease in the lattice thermal conductivity. The calculations of the unit cell sides are in good agreement with the experimental data^15^ as reported in Table S1 (Supplementary Information). The substitution of Y^3+^ with Lu^3+^ ions in YAG structure disrupts the perfect symmetry of the Y^3+^ ions coordination environment, leading to a modified crystal field symmetry. Also in this case, the structural variations are attributed to the difference in radius between Lu^3+^ ions (85 pm) and Y^3+^ ions (89 pm), leading to a contraction of the unit cell and the formation of point defects. There are two types of dodecahedral Y-O (Lu-O) bonds which are distinguished with subscripts 1 and 2 (see Table 3). Furthermore, the ions Al^3+^ occupy both octahedral (Al_1_) and tetrahedral (Al_2_) sites.

**Table 3:** Bond distances (after geometry optimization) for the two types of dodecahedral Y–O (Lu-O) bonds which can be identified with subscripts 1 and 2. Al^3+^ ions can occupy both octahedral (Al_1_) and tetrahedral (Al_2_) sites.

Bond length	Lu_1_–O	Lu_2_–O	Y_1_–O	Y_2_–O	Al_1_–O	Al_2_-O
YAG	0.000	0.000	2.302	2.407	1.928	1.781
Lu_33_	2.276	2.383	2.300	2.397	1.925	1.780
Lu_50_	2.277	2.380	2.299	2.391	1.924	1.779
Lu_67_	2.279	2.373	2.298	2.389	1.924	1.779
LuAG	2.280	2.370	0.000	0.000	1.923	1.778

### Determination of elastic constants

We calculated the elastic constants of Lu^3+^-doped mixed Y_3_Al_5_O_12_/ Lu_3_Al_5_O_12_ crystals by using the same computational framework.

For the prediction of thermal conductivity of Y_3_Al_5_O_12_ and Lu_3_Al_5_O_12_ pure crystals we calculated the mechanical properties reported in Table 4.

**Table 4:** Mechanical properties of Y_3_Al_5_O_12_ and Lu_3_Al_5_O_12_ pure crystals expressed in GPa.

C_ij_	Y_3_Al_5_O_12_	Lu_3_Al_5_O_12_
C_11_	330	346
C_12_	117	112
C_44_	105	114
B	188	191
G	106	115
E	266	284
υ	0.26	0.25

The experimental room-temperature values of Y_3_Al_5_O_12_ single-crystal elastic constants (C_11_ = 334 GPa, C_12_ = 111.2 GPa, and C_44_ = 115.1 GPa), bulk modulus (B = 185 GPa), shear modulus (G = 114 GPa), Young modulus (E = 283 GPa) and Poisson ratio (υ = 0.25) reported in ref.^52^ are in quite good agreement with our calculated data (see Table 5) for Y_3_Al_5_O_12_. As regard Lu_3_Al_5_O_12_ single-crystal, the reported data in Table 15 are in agreement with data reported in Ref.^53^ (C_11_ = 342 GPa, C_12_ = 112 GPa, and C_44_ = 115 GPa; B = 189 GPa, G = 115 GPa, E = 287 GPa and υ = 0.247). Table 5 reports the calculated structural and phonon parameters of the materials under study. These parameters were used to calculate the thermal conductivities of pure compounds (Y_3_Al_5_O_12_, Lu_3_Al_5_O_12_) by modified Slack equation.

**Table 5:** Structural and phonon parameters of the materials under study. The density, $\uprho$ is expressed in (kg/m^3^), ${\text{v}}_{\text{L}}$, ${\text{v}}_{\text{S}}$ and ${\text{v}}_{\text{a}}$ are expressed in (m/s), ${\theta }_{D}$ and ${\uptheta }_{a}$ are expressed in *K* and $\upgamma$ is dimensionless.

Ceramics	$\uprho$	${\text{v}}_{\text{L}}$	${\text{v}}_{\text{S}}$	${\text{v}}_{\text{a}}$	${\uptheta }_{D}$	${\uptheta }_{a}$	$\upgamma$
Y_3_Al_5_O_12_	4576	8477	4804	5341	720.129	167.127	1.567
Lu_3_Al_5_O_12_	6719	7151	4141	4596	624.32	144.89	1.490

#### Thermal conductivities models

Through the revised Slack equation, we obtained thermal conductivities in quite alignment with experimental values as reported in Table 6.

**Table 6:** Experimental and calculated thermal conductivities (Wm^−1^K^−1^) for Y_3_Al_5_O_12_ and Lu_3_Al_5_O_12_.

Compounds	Exp. data	Revised slack
Y_3_Al_5_O_12_	12.900^15^	10.623
Lu_3_Al_5_O_12_	9.600^15^	8.924

To evaluate the effects of increasing Lu^3+^ content in the samples Lu_33_, Lu_50_, Lu_67_, we used for ${k}_{0}$ the thermal conductivity of YAG and for ${k}_{100}$ that of LuAG. For the calculation of the corrective term $\Gamma$ (Eq. 24) we set ${c}_{i}$ as percent fraction of the i-*th* compound (*i* = LuAG or YAG), ${M}_{i}$ as the molar mass of the i-*th* compound and $\frac{{R}_{i}-R}{R}$ was replaced with $\frac{{R}_{i}-{R}_{mx}}{{R}_{mx}}$, where *R*_*i*_ is the average ionic radius in the i-*th* compound, and R_mx_ is the average ionic radius in mixed ceramic under study. In Table 7, we report the calculated distortion paramaters ${d}_{YAG}$ and ${d}_{LuAG}$ (Eq. 24), the average bond length ($\sigma$) and the average cell side length ($a$).

**Table 7:** ${d}_{YAG}$ and ${d}_{LuAG}$ are the distortions parameters calculated with respect to YAG and LuAG. $a$ is the unit cell side average value, σ is the mean bond length. All the reported quantities are reported in Å.

Compound	${d}_{YAG}$	${d}_{LuAG}$	$a$	σ
YAG	0	0.110	11.989	2.105
Lu_33_	0.043	0.071	11.958	2.098
Lu_50_	0.061	0.060	11.945	2.094
Lu_67_	0.075	0.043	11.930	2.091
LuAG	0,110	0	11.898	2.088

Table 8 reports the thermal conductivity values calculated with Klemens Eq. (19) and modified Klemens Eq. (26) compared with experimental data. In Eq. 26 we set $a=-0.00505$ and $r =-0.11348$. We indicate with Mod.${{k}_{L}}_{\text{K}}(d)$ the revised Klemens equation with YAG and LuAG experimental thermal conductivities and rev.${{k}_{L}}_{\text{K}}(d)$ the revised Klemens equation with YAG and LuAG thermal conductivities calculated by revised Slack equation.

**Table 8:** Experimental and calculated thermal conductivities (expressed in Wm^−1^ K^−1^).

Compound	${{k}_{L}}_{K}$	Mod.${{k}_{L}}_{\text{K}}\left(d\right)$	Rev.${{k}_{L}}_{\text{K}}\left(d\right)$	Experimental
YAG	12.900	12.900	10.623	12.900^15^
Lu_33_	7.223	7.724	6.858	7.800^15^
Lu_50_	6.985	7.449	6.707	7.500^15^
Lu_67_	7.178	7.747	7.055	7.600^15^
LuAG	9.600	9.600	8.924	9.600^15^

**Figure 5:**
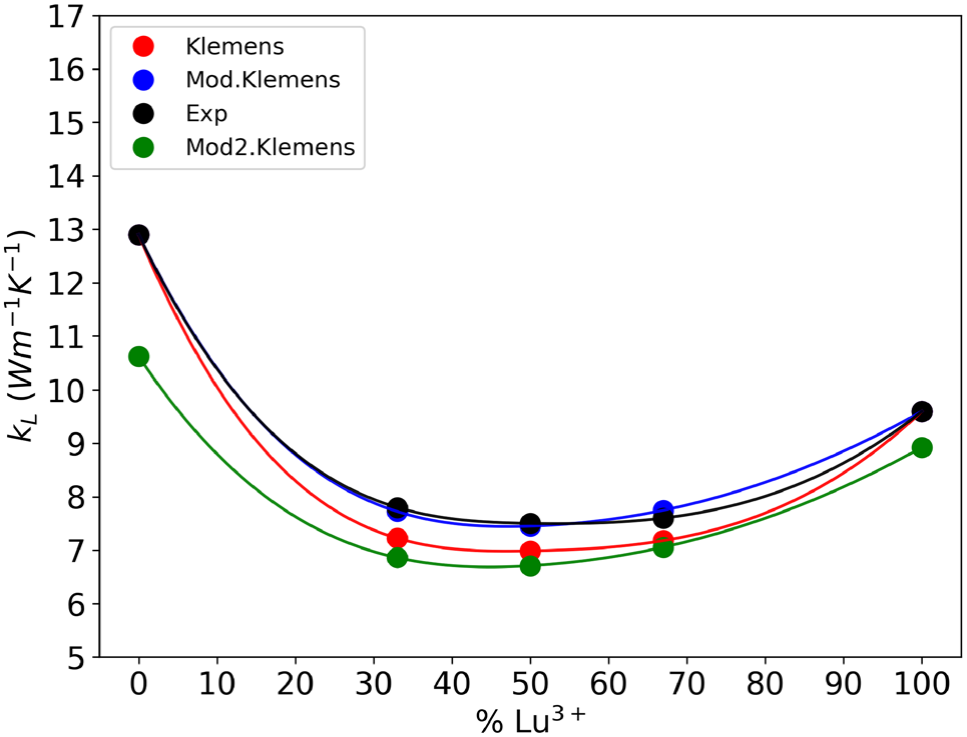
Experimental (black) and calculated (blue, red and green) values of thermal conductivity for the entire set of samples.

It can be seen from the data of Table 8 and Fig. 5 that Mod.${{k}_{L}}_{\text{K}}$ equation provides a more accurate evaluation of the thermal conductivity than the original Klemens model. Regarding the method rev.${{k}_{L}}_{\text{K}}$, its accuracy is apparently lower, but we have to take into account that in this case both the original Klemens model and Mod.${{k}_{L}}_{\text{K}}$ are seeded with the experimental data of thermal conductivity of YAG and LuAG, while in rev.${{k}_{L}}_{\text{K}}$ relies on thermal conductivity values calculated from the elastic constants. We want to point out that, while the evaluation provided by rev.${{k}_{L}}_{\text{K}}$ cannot compete in accuracy with the other two, this evaluation scheme can be used even when the thermal conductivity data of the pure compounds are not know, as long as the elastic constants and lattice parameters are available.

The statistical analysis of the data reported in Table 8 highlights the performance of each model. In particular we obtained a Percentage Relative Error of 6.606% for the original Klemens equation, 1.196% for Mod.${{k}_{L}}_{\text{K}}$ and 9.940% for rev.${{k}_{L}}_{\text{K}}$. This data shows a significant accuracy increase of our Mod.${{k}_{L}}_{\text{K}}$. For the rev.${{k}_{L}}_{\text{K}}$ it must be taken into account that this model is affected by the errors of both revised Klemens and revised Slack equations.

### Prediction of thermal conductivity in 5 at.% Tm^3+-^doped (Y,Sc)_2_O_3_ transparent ceramics

#### Calculation of the lattice parameters

**Figure 6:**
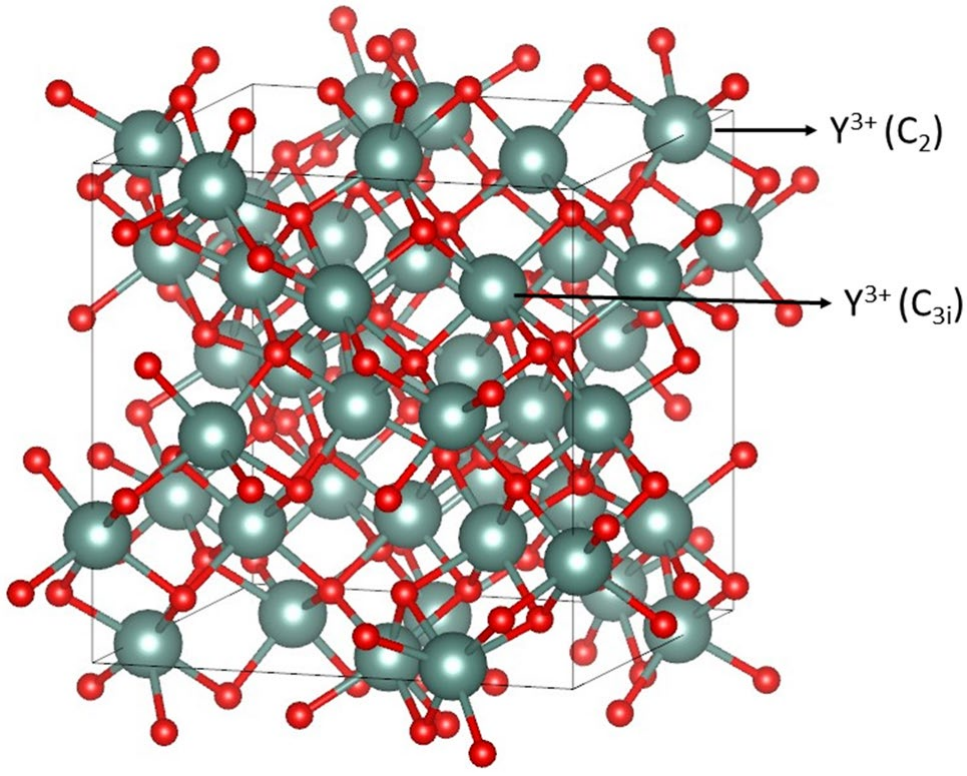
Lattice structure of Y_2_O_3._ Yttrium sites with symmetry C_3i_ and C_2_ are colored in dark green. The symmetry of the sites is reported in round brackets.

To assess the accuracy of the PBEsol functional with Grimme D3 dispersion correction, we compared the modeled structural parameters of the pure Y_2_O_3_ crystal to the experimental data reported in^54^. This data served as an established benchmark against which the predictive power of the functional could be evaluated. Y_2_O_3_ exhibits a body-centered cubic lattice structure (see Fig. 6) with the space group $Ia\overline{3 }$ and Y^3+^ sites with symmetry C_3i_ (Y_1_) and C_2_ (Y_2_), and O^2-^ ions without symmetry (O).

The PBEsol functional with Grimme D3 dispersion correction produced a cubic unit cell with a lattice side of 10.61 Å, which is in agreement with experimental data^54^. In particular, the mean bond lengths of the three different Y_1_-O bonds in the C_3i_ sites were determined to be 2.33 Å, 2.27 Å, and 2.24 Å, while the mean bond length of the Y_2_-O bonds in the C_2_ sites was 2.28 Å. These results suggest that the PBEsol functional with Grimme D3 dispersion correction is a reliable tool for accurately modelling the structural properties of Y_2_O_3_ crystals and other related materials.

In a preliminary investigation, we performed DFT calculations with PBEsol functional on 5*at.%*Tm:Y_2_O_3_. The Tm^3+^ cations replace Y^3+^ at both C_2_ (Y_2_) and C_3i_ (Y_1_) sites. We observed very small perturbations in lattice cell parameters (< 0.1%), bonds lengths and inter-cationic distances (<1%). This result was not unexpected as Tm^3+^ cations have an ionic radius (85.8 pm) very similar to the ionic radius of Y^3+^ (89.3 pm).

To reduce computational time while maintaining structural accuracy, we utilized a structural relaxation technique wherein the unit cell angles were fixed at 90°. This approach assumes that deviations from a perfect cubic lattice are negligible, thus allowing for an efficient optimization of the atomic positions within the crystal structure. This assumption is supported by the experimental data reported in^55^.

The lattice vector values obtained from the structural relaxation of 5at.%Tm:Y_2_O_3_ are shown in Table 9.

**Table 9:** Sides (a, b, c) angles (α, β, γ) and volume (V) of the 5 at.%Tm:Y_2_O_3_ unit cell compared with experimental structural parameters^55^. All sides of the cell are given in Å, the volume in Å^3^ and the angles in degrees. We also report the percentage relative deviation (Δ%) of the calculated data from the experimental values.

Unit cell	a = b = c	α = β = γ	V
exp^55^	10.5989	90°	1190.6
PBEsol	10.5960	90°	1189.7
Δ%	0.0270%	0%	0.08%

In Table 10 the X_1_-O experimental^54^ and calculated average values of bond lengths are reported. X_1_ is referred to Y^3+^ in Y_1_ site for experimental data^54^ and to Tm^3+^ which replaced Y^3+^ in Y_1_ site for PBEsol calculated data.

**Table 10:** Bond lengths X_1_-O, X_2_-O, angle {X_1_OY_2_} and percentage of the relative deviation (Δ%) of the calculated data from the experimental values. More precisely, X_1_-O refers to 3 different types of bond lengths within the C_2_ site. All bond lengths are given in Å and the angles in degrees.

Bond length	X_1_–O	X_1_–O	X_1_-O	{X_1_OY_2_}
exp^54^, Y_2_O_3_	2.33326	2.27212	2.24472	124.16
PBEsol—Tm:Y_2_O_3_	2.32927	2.27731	2.23223	124.49
Δ%	0.17101%	0.66854%	0.55642%	0.02%

Looking at Table 10 a slight contraction of two bond lengths can be observed when Tm^3+^ replaced Y^3+^ in the Y_1_ site. However, the bond lengths variations are smaller than 1% and this is principally due to the similar ionic radius between Y^3+^ and Tm^3+^. PBEsol functional have been used also to study the structural variations when an increasing concentration of Sc^3+^ is added to the 5 at.%Tm:Y_2_O_3_ sample. When Y^3+^ is substituted with Sc^3+^ a notable contraction of both the cell parameters and of the bond lengths (inter-cationic distances) is observed. This is mainly due to the differences in ion radius between Y^3+^ and Sc^3+^ as the ionic radius of Sc^3+^ (i.e. 74.5 pm) is 19% smaller than Y^3+^ (i.e. 89.3 pm). Due to the complexity of these ternary mixture sesquioxides, in this case, we performed a more accurate structural analysis of DFT output going to deeply inspect local symmetry losses through the Pair Distribution Function (PDF) and X-Ray Diffraction (XRD) simulations (see Fig. S1, Fig. S2 and Fig. S3 in Supplementary Information). The reported data refer to 5at.%Tm:Y_2_O_3_^55^, Sc_12_, Sc_25_ and Sc_50_. The calculations are carried out with 50% Sc^3+^ sample (and not with 49.8% Sc^3+^ as in the experiment) only for practical convenience in the cell construction.

The increasing concentration of Sc^3+^ results in a contraction of the lattice vectors and, in turn, of the volume (see Table 11). The trends obtained by DFT calculations were compared with Vegard’s law^21^. The lattice side of 5at.%Tm:Y_2_O_3_ is ${{\varvec{a}}}_{5\mathbf{a}\mathbf{t}.\mathbf{\%}\mathbf{T}\mathbf{m}:\mathbf{Y}2\mathbf{O}3}$ =10.60 Å while the lattice side of Sc_2_O_3_ is ${\varvec{a}}$_Sc2O3_ = 9.79 Å^56^. By using the Vegard’s law, we have:

**Table 11:** Sides (**a**, **b**, **c**), angles (**α**, **β**, **γ)** and volume (**V**) of the reference unit cell^55^ compared with calculated unit cell parameters of Sc_12_, Sc_25_ and Sc_50_ samples.

Cell	a	b	c	α	β	γ	V
exp^55^	10.5989	10.5989	10.5989	90.0°	90.0°	90.0°	1190.6
Sc_12_	10.5151	10.5242	10.5199	89.8°	90.2°	89.9°	1164.1
Sc_25_	10.4227	10.4249	10.4293	89.7°	90.2°	90.1°	1133.2
Sc_50_	10.2225	10.2238	10.2222	90.1°	90.1	89.9°	1074.6

$$a\text{Sc}12= 0.12a\text{Sc}2\text{O}3 + (1-0.12) \cdot {a}_{5\text{at}.\text{\%Tm}:\text{Y}2\text{O}3 }= 10.51\text{ \AA }$$

$$a\text{Sc}25= 0.25 \cdot a\text{Sc}2\text{O}3 + (1-0.25) \cdot {a}_{5\text{at}.\text{\%Tm}:\text{Y}2\text{O}3 } = 10.41\text{ \AA }$$

$$a\text{Sc}50=0.50\cdot a\text{Sc}2\text{O}3+ (1-0.50) \cdot {a}_{5\text{at}.\text{\%Tm}:\text{Y}2\text{O}3 } = 10.20\text{ \AA }$$

The agreement between the lattice values calculated with Vegard’s law, the ones calculated at DFT theory level and experimental values is very good, see Table 12.

**Table 12:** Side ${\varvec{a}}$ values calculated with Vegard’s law and the mean values of the lattice side calculated with PBEsol functional both compared with experimental data. All values are expressed in Å.

System	Vegard’s law	PBEsol	$exp$
$\large {a}_{5\text{at}.\text{\%Tm}:\text{Y}2\text{O}3}$	10.60	10.60	10.60
$\large a$_Sc12_	10.51	10.52	10.50
$\large a$_Sc25_	10.41	10.43	10.40
$\large a$_Sc50_	10.20	10.22	10.22

The increase of the concentration of Sc^3+^ increase the entropy of the system due to the formation of new Sc-O bonds, which add new configurations to the system and increase the number of possible arrangements of the atoms (see Fig. S1); in other words, the number of complexions rises. Accordingly, the partial loss of symmetry of the C_3i_ and C_2_ sites in Sc-doped 5at.%Tm:Y_2_O_3_ can be attributed to the atomic-scale distortions caused by the Sc^3+^ ions. The substitution of Y^3+^ with Sc^3+^ introduces local perturbations in the crystal lattice structure, which lead to a decrease in the crystal field symmetry around these sites, resulting in a lower degree of the symmetry coordination with the surrounding oxygen atoms. Three types of bond lengths in the doped crystal are possible, see Table 13.

**Table 13:** Average bond lengths Sc–O, X–O and Y–O (X can be Y^3+^ or Sc^3+^). These data are extracted from PBEsol calculations and are expressed in Å.

Bond lengths	Sc–O	X–O	Y–O
Sc_12_	2.153	2.252	2.335
Sc_25_	2.130	2.251	2.330
Sc_50_	2.126	2.223	2.305

It is clearly observed a local change of the structure with increasing Sc^3+^ concentration through the X-ray atomic PDF; the latter is an X-ray scattering technique which can be used to study the local structure of materials on an atomic scale. It is based on the concept of radial distribution function, which measures the probability of finding an atom at a certain distance from another atom. By analyzing the PDF output, it is possible to extract information on the average interatomic distances, bond lengths, bond angles, coordination numbers, as well as local symmetry of the material. We simulated PDF with EXPO^57^, using as input the optimized structures of 5at.%Tm:Y_2_O_3_, Sc_12_, Sc_25_ and Sc_50_ because PDF is a powerful tool for probing the structural properties of crystalline and amorphous materials. In our calculation PDF is used to describe the distribution of ion pairs (Y1-O, Y2-O, X-O, Sc-O) within the volume occupied by the system.

We show how the system partially loses the recognition of the C_3i_ and C_2_ sites by increasing the concentration of Sc^3+^ leading to an intensification of the formation probability of the Sc-O bond with length in the interval 2.126–2.153 Å (see Fig. S1).

Accordingly, the calculated XRD patterns of the samples Y_2_O_3_ and Sc_50_ show the shift of the most intense peaks and the appearance of new ones (see Fig. S2).

The comparison between the experimental^58^ and calculated spectra of the Sc_50_ sample (see Fig. S3) confirms the good reliability of the structural data calculated with PBEsol which returns an accurate picture of the local disorder introduced by the Sc^3+^ cations as well as the effect of contraction of the cell parameters.

#### Determination of elastic constants

By using the same computational framework^16^ we calculated the elastic constants of Tm^3+^ doped Y_2_O_3_, Tm^3+^ doped Sc_2_O_3_ and Tm^3+^ doped, mixed Y_2_O_3_ and Sc_2_O_3_.

The experimental room-temperature values of single-crystal (Y_2_O_3_) elastic constants (C_11_ = 223.6 GPa, C_12_ = 112.4 GPa, and C_44_ = 74.6 GPa), bulk modulus (B = 149.5 GPa), shear modulus (G = 66.3 GPa), Young modulus (E = 173.0 GPa) and Poisson ratio (υ = 0.307) reported in Ref.^59^ are in good agreement with our calculated data (see Table 14) for 5at.%Tm:Y_2_O_3_; moreover, they are also comparable with data reported in Ref.^60^ (B = 153.8, G = 62.4, E = 165.0 and υ = 0.320). Table 15 summarizes the calculated structural and phonon parameters of the materials under study. It is worth to note these parameters will be used to calculate the thermal conductivities of pure compounds (Y_2_O_3_, Sc_2_O_3_) by modified Slack equation.

**Table 14:** Mechanical properties of 5 at.% Tm^3+^-doped Y_2_O_3_, Sc_2_O_3_, Sc_12_, Sc_25_, Sc_50_ (see section Ab initio calculations of elastic constants), expressed in GPa, with the exception of υ, which is a dimensionless number.

C_ij_	5 at.% Tm:Y_2_O_3_	Sc_12_	Sc_25_	Sc_50_	5 at.% Tm:Sc_2_O_3_
C_11_	221.58	224.12	228.96	240.84	277.30
C_12_	114.32	115.14	116.28	119.14	125.88
C_44_	72.21	71.99	72.57	75.32	88.52
B	150.07	151.47	153.84	159.71	176.35
G	64.10	64.39	65.57	69.15	83.15
E	167.33	169.l9	171.49	181.30	215.57
υ	0.313	0.314	0.315	0.311	0.296

**Table 15:** Structural and phonon parameters of the materials under study. $\uprho$ is expressed in (kg/m^3+^), ${\text{v}}_{\text{L}}$, ${\text{v}}_{\text{S}}$ and ${\text{v}}_{\text{a}}$ are expressed in (*m/s*), ${\theta }_{D}$ and ${\uptheta }_{a}$ are expressed in *K* and $\upgamma$ is dimensionless.

Ceramics	$\uprho$	${\text{v}}_{\text{L}}$	${\text{v}}_{\text{S}}$	${\text{v}}_{\text{a}}$	${\uptheta }_{D}$	${\uptheta }_{a}$	$\upgamma$
Y_2_O_3_	5021	6849	3573	3998	483.28	141.31	1.815
5at.%Tm:Y_2_O_3_	5245	6701	3523	3942	473.24	138.38	1.825
Sc_12_	5144	6793	3538	3959	482.79	141.32	1.832
Sc_25_	5016	6933	3606	4035	497.10	145.35	1.837
Sc_50_	4746	7286	3817	4270	535.41	156.56	1.842
Sc_2_O_3_	3819	8672	4666	5210	677.60	198.13	1.750

#### Thermal conductivities models

The results of the thermal conductivities of pure Yttria and Scandia calculated by using the modified Slack equation (see Eq. 16) shows a good agreement with the experimental data^36,61^. In Table 16 the experimental and calculated thermal conductivities of the pure samples are reported.

**Table 16:** Experimental and calculated thermal conductivities (expressed in Wm^−1^ K^−1^) for Y_2_O_3_ and Sc_2_O_3_.

Compounds	Exp. data	Revised slack
Y_2_O_3_	12.720^35^	12.084
Sc_2_O_3_	17.000^36^	16.822

Hereon, ${k}_{0}$ and ${k}_{100}$ will label the thermal conductivities of Y_2_O_3_ and Sc_2_O_3_ as reported in Table 16. The thermal conductivity characteristics of 5*at.%* Tm^3+^-doped (Y,Sc)_2_O_3_ transparent ceramics present a more intricate scenario compared to those of Lu-doped Yttrium Aluminum Garnet. As a matter of fact, in Sc_12_, Sc_25_ and Sc_50_ three compounds are involved, contrasting with the simpler binary systems in Lu-doped Yttrium Aluminum Garnet. We utilize a value of *a* = 0.027855 and *r* = -0.11352. For the calculation of the corrective term $\Gamma$ (see Eq. 25) ${c}_{i}$ was set as percent fraction of dopant, ${M}_{i}$ as the molar mass of the dopant and $\frac{{R}_{i}-R}{R}$ was replaced with $\frac{{R}_{i}-{R}_{mx}}{{R}_{mx}}$, where ${R}_{i}$ is the average radius of the ions in the host, ${R}_{mx}$ is the average radius of the ions in the mixed ceramic under study. In Table 17, the distortion parameters ${d}_{Y2O3}$, ${d}_{Sc2O3}$ and ${d}_{Tm2O3}$ (calculated according to Eq. 24), the average bond length ($\sigma$) and the average cell side length ($a$) are listed.

**Table 17:** Structural parameters:${d}_{Y2O3}$, ${d}_{Sc2O3}$ and ${d}_{Tm2O3}$ are the distortion parameters with respect to undoped hosts (Y_2_O_3_ and Sc_2_O_3_); $a$ is the unit cell side average value and σ is the mean bond lengths. All the values are reported in Å.

Compound	${d}_{Y2O3}$	${d}_{Sc2O3}$	${d}_{Tm2O3}$	$a$	σ
Y_2_O_3_	0.000	0.610	0.019	10.6056	2.274
5at.%Tm:Y_2_O_3_	0.005	0.592	0.008	10.5960	2.263
Sc_12_	0.126	0.542	0.089	10.5197	2.247
Sc_25_	0.206	0.468	0.145	10.4256	2.237
Sc_50_	0.349	0.328	0.259	10.2428	2.218
Sc_2_O_3_	0.613	0.000	0.524	9.8630	2.126

Table 18 reports the thermal conductivity values calculated by Klemens equation (see Eq. 19) and modified Klemens equation (see Eq. 26) compared with experimental data. With *d* in round parenthesis, we identify the revised Klemens results. To calculate the Eq. 26$a=0.027858$ and $r=-0.113485$ were set.

**Table 18:** Experimental and calculated values of thermal conductivity (expressed in Wm^−1^ K^−1^).

Compounds	${{k}_{L}}_{\text{K}}$	Mod.${{k}_{L}}_{\text{K}}(d)$	rev.${{k}_{L}}_{\text{K}}(d)$	Experimental
Y_2_O_3_	12.720^35^	12.720^35^	12.084	12.720^35^
5at.%Tm:Y_2_O_3_	8.470	5.964	5.77	5.947^61^
Sc_12_	5.731	5.087	4.955	5.179^61^
Sc_25_	4.925	4.562	4.465	4.531^61^
Sc_50_	4.532	4.300	4.236	4.300^58^
Sc_2_O_3_	17.000^36^	17.000^36^	16.822	17.000^36^

Mod.${{k}_{L}}_{\text{K}}(d)$ refers to revised Klemens equation with Y_2_O_3_ and Sc_2_O_3_ experimental thermal conductivities and rev.${{k}_{L}}_{\text{K}}(d)$ refers to revised Klemens equation with Y_2_O_3_ and Sc_2_O_3_ thermal conductivities calculated by revised Slack equation.

**Figure 7:**
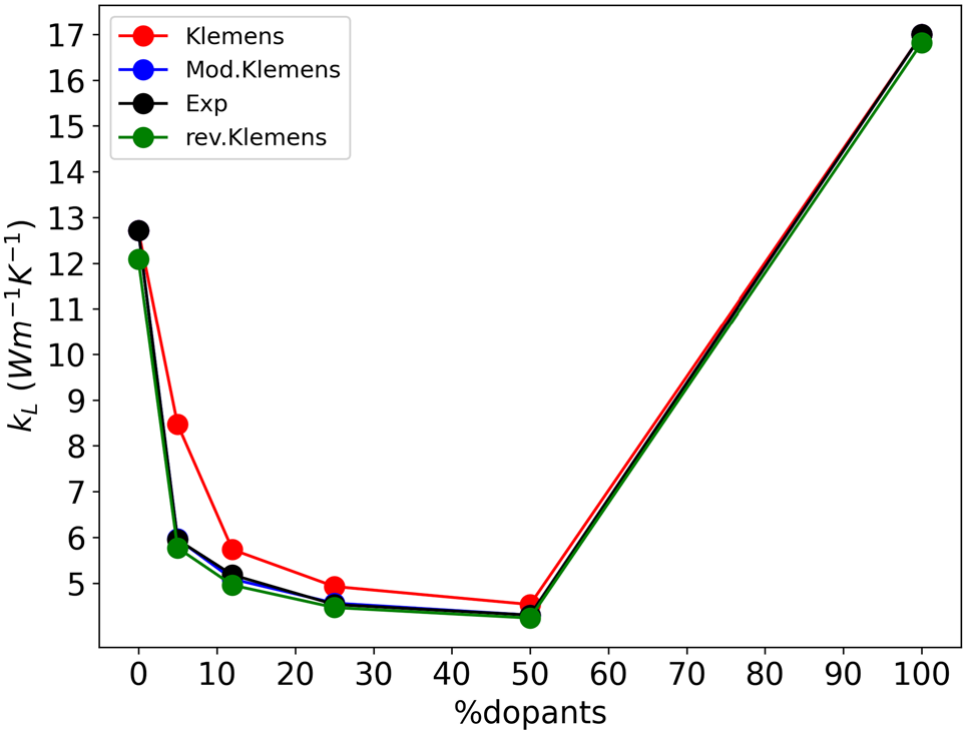
Experimental (black) and calculated (red, blue and green) values of thermal conductivity for the entire set of samples based on 5% Tm^3+^ doped mixed Y_2_O_3_/Sc_2_O_3_ ceramics.

Figure 7 report the comparison between experimental and calculated values of thermal conductivity obtained for 5 at.% Tm^3+^ doped mixed Y_2_O_3_/Sc_2_O_3_ ceramics.

The modified Klemens and original Klemens models show trends in good agreement with the experimental data^61^. Anyway both the proposed Mod.Klemens and rev.Klemens method show the best agreement with respect to the experimental trend. In our vision, the introduction of the parameter *d* in the model allows a more accurate description of these mixed composition compounds because it takes into account the lattice deformation due to the point defects. This is further confirmed by the statistical analysis of the data reported in Table 18. The estimation of the Percentage Relative for the original Klemens equation is 16.793% which is significantly higher with respect to Mod.${{k}_{L}}_{\text{K}}$ (0.687%) and rev.${{k}_{L}}_{\text{K}}$ (2.562%). We can confirm that our implemented method allows a more accurate prediction of thermal conductivity in this class of materials in comparison with the respective classical models.

## Conclusions

This study introduces a computational framework designed to estimate the thermal conductivity of optical materials, specifically targeting cubic oxides. Our methodology encompasses a series of comprehensive steps utilizing DFT calculations to extract essential structural parameters and elastic constants. These parameters constitute key inputs for refined the Slack and Klemens equations, enhancing the precision of lattice thermal conductivity predictions in these materials. Moreover, the above mentioned parameters can be also obtained from free on line materials database further shortening the time and the costs of the information achievement. Our approach involves the modification of the Slack equation by introducing a corrective factor of the Grüneisen parameter, i.e. ~ 0.3·γ^4^. Also, Klemens equation has been revised by the introduction of a distortion parameter, *d*, accounting for the impact of point defects on lattice symmetry, a critical factor significantly influencing the thermal conductivity.

The analysis of the outputs of our revised method in comparison with those of the classical Slack and Klemens equations gives rise to a substantial improvement in the accuracy of thermal conductivity prediction for the class of compound analyzed in this work.

Our model is intended to provide a good estimation of thermal conductivity of poorly characterized materials starting from experimental data that are more readily available than thermal conductivity itself, such as lattice parameters and mechanical properties. Thermal properties of the analysed class of materials are particularly important for optical applications and specifically for candidate laser hosts. Further investigations addressed on testing the proposed model also on materials belonging to different space group symmetry are in progress.

### Supplementary Information

Supplementary Information.

## Data Availability

We implemented a repository on GitHub containing the worksheets used for the calculations reported in the paper (https://github.com/BabbyMatisse/Thermal-Conductivity-).
