# Supplementary material for: Cost-effective method for computational prediction of thermal conductivity in optical materials based on cubic oxides
Source: Sci Rep. 2024 Jun 10;14:13343. doi: 10.1038/s41598-024-63302-6 (PMC11165009; doi:10.1038/s41598-024-63302-6)
Supplement: Supplementary file 1 — Supplementary Information. [file 41598_2024_63302_MOESM1_ESM.docx]

Supplementary Information

**Cost-Effective Method for Computational Prediction of Thermal Conductivity in Optical Materials based on Cubic Oxides**

A. Santonocito,^1,2^ B. Patrizi,^2,3*^ A. Pirri,^4,^ M. Vannini,^2^ G. Toci^2^

^1^ Dipartimento di Chimica, Università di Pisa, Via Giuseppe Moruzzi 13, Pisa, I-56124, Italy

^2^ Istituto Nazionale di Ottica, Consiglio Nazionale delle Ricerche, INO-CNR, Via Madonna del Piano 10, I-50019, Sesto Fiorentino, FI, Italy

^3^ European Laboratory for Non Linear Spectroscopy, LENS, Via Nello Carrara 1, I-50019, Sesto Fiorentino, FI, Italy

^4^ Istituto di Fisica Applicata “N. Carrara”, Consiglio Nazionale delle Ricerche, IFAC-CNR, Via Madonna del Piano 10, I-50019, Sesto Fiorentino, FI, Italy

***corresponding author:** [**barbara.patrizi@ino.cnr.it**](mailto:barbara.patrizi@ino.cnr.it)**;** [**patrizi@lens.unifi.it**](mailto:patrizi@lens.unifi.it)

Tab.S1: Sides a, b, c, angles α, β, γ, volume V and percentage composition $\boldsymbol{x}$ of (Lu_x_Y_1-x_)_3_Al_5_O_12_. The percentage relative deviation (Δ%) of the calculated data from the experimental are also reported. Cell sides are expressed in Å, cell volume in Å^3^ and the angles in degrees.

| Compound | %Lu^3+^ | Cell Sides a=b=c | Cell Angles α= β= γ | Cell Volume |
| --- | --- | --- | --- | --- |
| **Y_3_Al_5_O_12_** | **0** |  |  |  |
| **Exp** ^15^ |  | 12.0075 | 90° | 1731.24 |
| **PBEsol** |  | 11.9895 | 90° | 1723.48 |
| **Δ%** |  | 0.1497% | 0% | 0.45% |
| **Lu_33_** | **33.3** |  |  |  |
| **Exp** ^15^ |  | 11.9765 | 90° | 1717.87 |
| **PBEsol** |  | 11.9577 | 90° | 1709.79 |
| **Δ%** |  | 0.1569% | 0% | 0.47% |
| **Lu_50_** | **50** |  |  |  |
| **Exp** ^15^ |  | 11.9611 | 90° | 1709.70 |
| **PBEsol** |  | 11.9445 | 90° | 1704.13 |
| **Δ%** |  | 0.1388% | 0% | 0.326% |
| **Lu_67_** | **66.7** |  |  |  |
| **Exp** ^15^ |  | 11.9438 | 90° | 1703.53 |
| **PBEsol** |  | 11.9298 | 90° | 1696.18 |
| **Δ%** |  | 0.1172% | 0% | 0.43% |
| **Lu_3_Al_5_O_12_** | **100** |  |  |  |
| Exp ^15^  PBEsol  Δ% |  | 11.9164  11.8978  0.1561% | 90°  90°  0% | 1692.13  1684.22  0.46% |


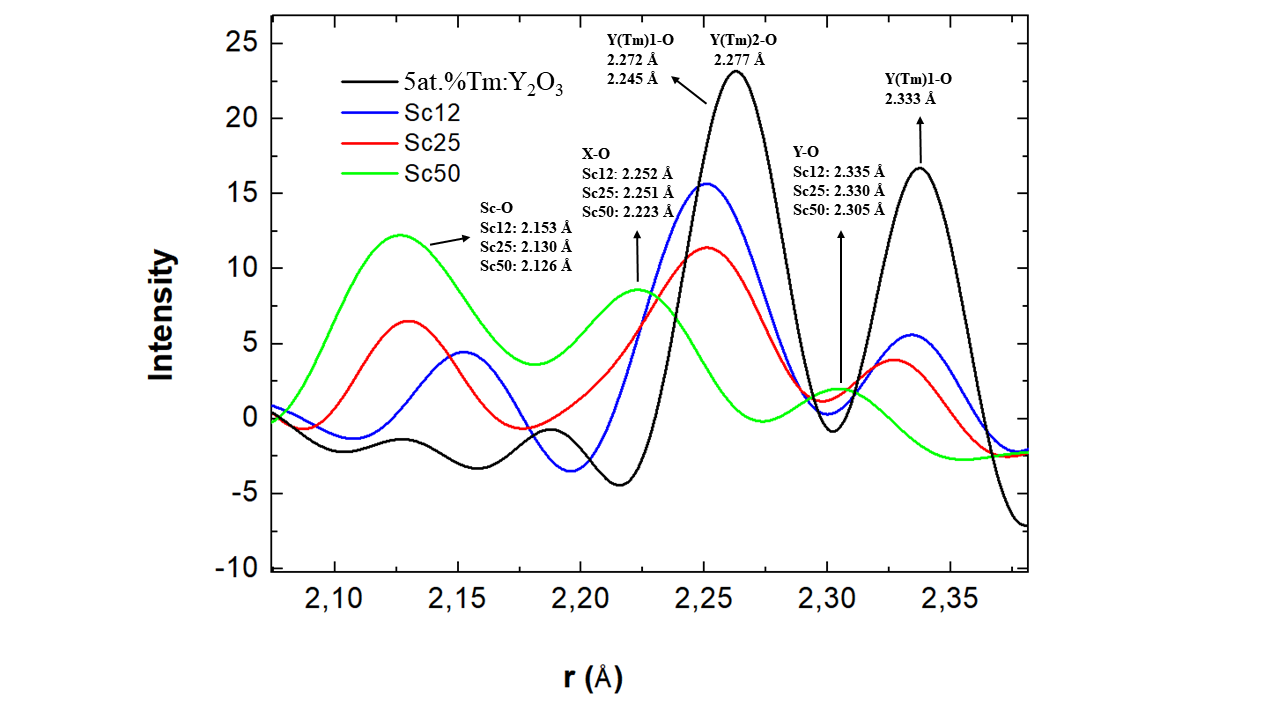


**Fig.S1**: Pair distribution function of the studied systems showing the increase of the entropy of the system due to the increasing Sc^3+^ concentration.


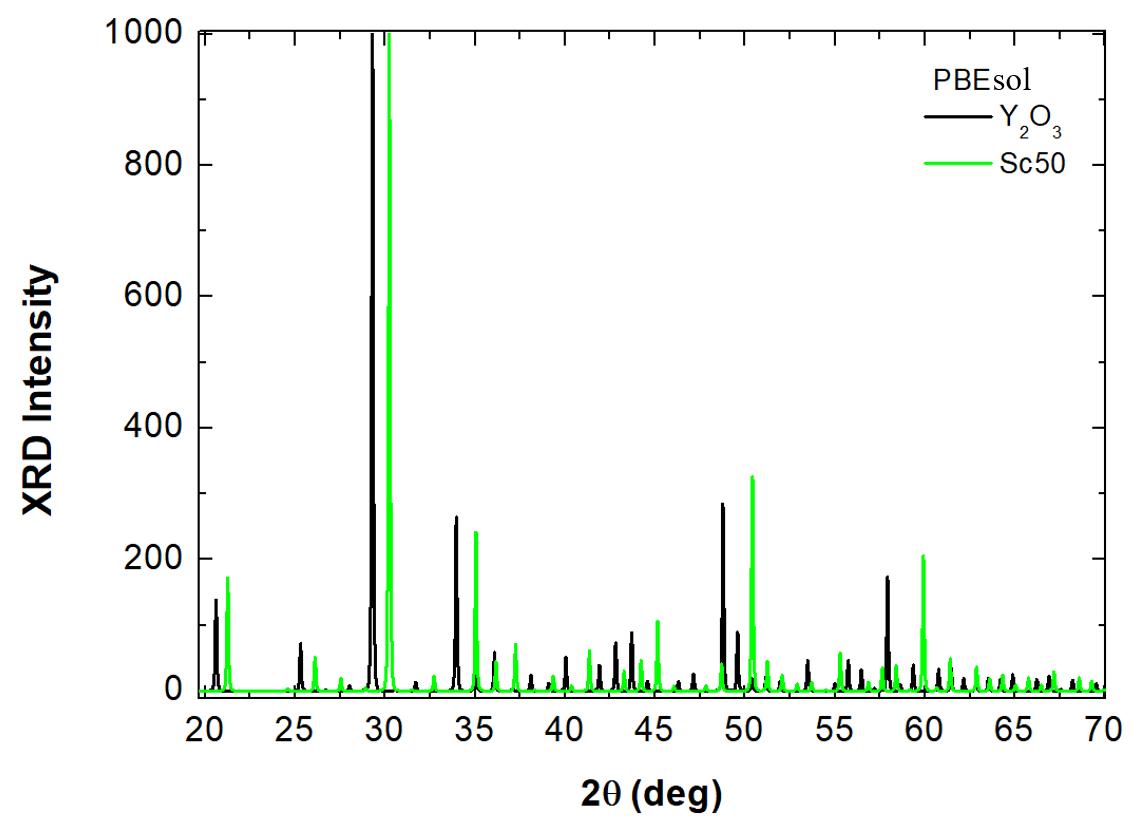


**Fig.S2**: Y_2_O_3_ and Sc_50_ XRD patterns extracted from the structure optimized with PBEsol. XRDs spectra were calculated with EXPO^1^.


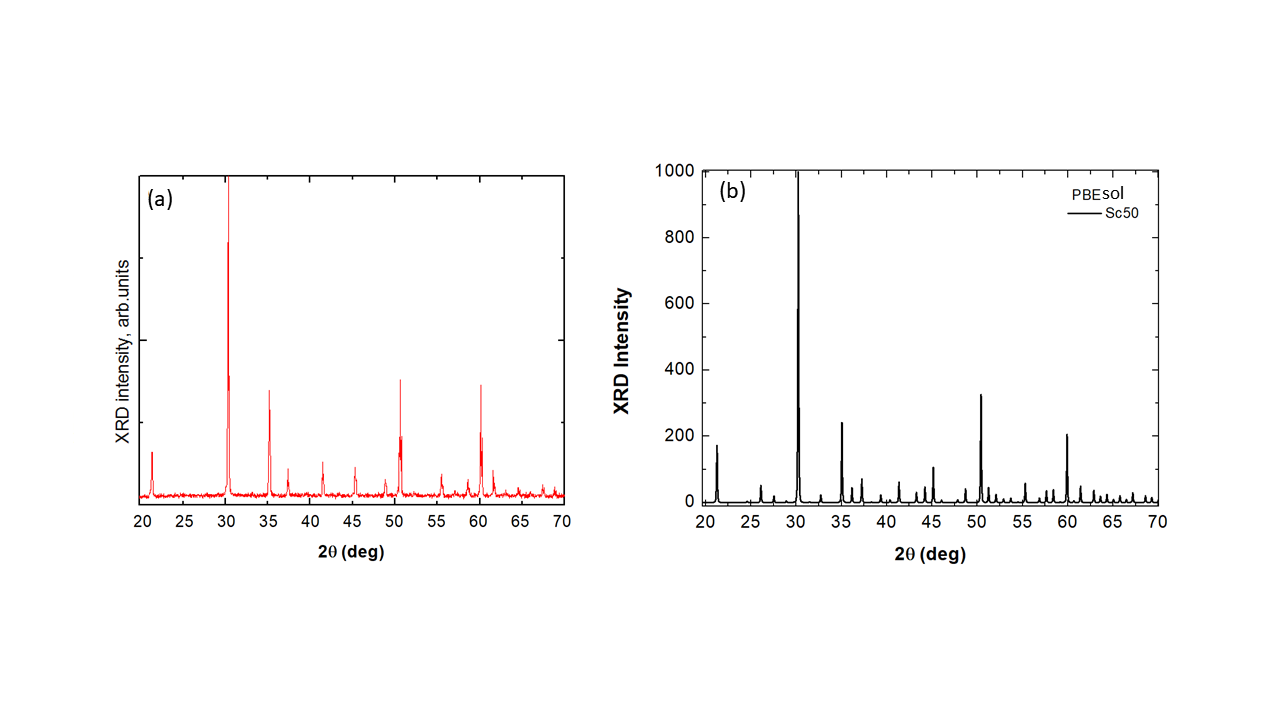


**Fig.S3**: Comparison between (a) Experimental Sc50 XRD spectrum (from ref. ^2^) and (b) Sc50 XRD spectrum extracted from the structure optimized with PBEsol and calculated with EXPO^1^.

**References**

1. Altomare, A., Ciriaco, F., Cuocci, C., Falcicchio, A. & Fanelli, F. Combined powder X-ray diffraction data and quantum-chemical calculations in EXPO2014. *Powder Diffr.* **32**, S123–S128 (2017).
2. Maksimov V. V. et al. Structural, Spectroscopic and Laser Properties of Transparent Tm:Ysco3 Ceramic Based on Gas-Phase Synthesised Nanoparticles. *Opt. Mater. (Amst).* (just accepted) (2024) doi:http://dx.doi.org/10.2139/ssrn.4639279.
